# The complete mitochondrial genome of *Glischrochilus (Librodor) japonius* (Coleoptera: Nitidulidae)

**DOI:** 10.1080/23802359.2025.2606449

**Published:** 2025-12-24

**Authors:** Huicong Du, Zi Yang, Wenping Luo, Jiahui Gu, Hongyu Luo, Yili Di, Jingyuan Wang, Jingxin Shen

**Affiliations:** ^a^ Jiangsu Provincial Key Laboratory of Coastal Wetland Bioresources and Environmental Protection, School of Wetlands, Yancheng Teachers University, Yancheng, China; ^b^ Shandong Provincial Yantai Ecological Environment Monitoring Center, Yantai, China; ^c^ School of Hydraulic Engineering, Fujian College of Water Conservancy and Electric Power, Yong’an, China

**Keywords:** Mitogenome, phylogenetic status, sap beetle

## Abstract

*Glischrochilus japonius* is a type of forestry pest that causes damage to various garden plants. In this study, the complete mitogenome of *G. japonius* was sequenced for the first time., The circular mitogenome comprises of 15,357 bp with an AT content of 77.51%, including 13 protein-coding genes (PCGs), 22 tRNA genes, 2 rRNA genes and one control region. The genera of the analyzed beetles from Nitidulidae in phylogenetic relationships are monophyletic. The mitochondrial genome structure of *G. japonius* is stable and conformed, and resolving the phylogenetic relationships amongst the sap beetles requires continued accumulation and improvement of mitogenome data.

## 1. Introduction

The sap beetle *Glischrochilus (Librodor) japonius* (Motschulsky, 1857), belonging to the family Nitidulidae under the order Coleoptera, is widely distributed in China. The length of adult is about 7–14 mm, its elytra have four yellow to red serrated markings, and it mainly attacks ornamental plants such as willow, poplar and camellia etc., (Li et al. 2025). The adults and larvae of *G. japonius* can be found in the wounds, cracks and sap of trees, feeding on plant sap, which weakens the nutrient supply of these host plants and affects their growth and development (Guo et al. 2016). Existing research on *G. japonius* has mainly focused on morphological classification (Lee et al. 2020), living habits (Okada and Miyatake 2007), and intraspecific competition (Okada et al. 2008). However, due to the scarcity of molecular data for species classified as Nitidulidae, the phylogenetic status of this sap beetle remains unclear (Xu et al. 2021). In recent years, complete mitogenomes have been widely used as molecular markers in studies of insect phylogenetic relationships and phylogeography, owing to their characteristics of maternal inheritance and rapid evolution (Du et al. 2021). To date, only approximately 40 sequences of species belonging to the Nitidulidae family in NCBI, and many of these sequences have not been verified and contain duplicates or errors. Therefore, this study provides a detailed annotation and analysis of the mitogenome sequence of *G. japonius*, and combined with existing mitochondrial data in NCBI, conducts a phylogenetic analysis of the species within the Nitidulidae. These findings will provide a theoretical basis for further clarifying the phylogenetic status of sap beetles.

## 2. Materials and methods

### 2.1. Sampling and mitochondrial genome sequencing analysis

*Glischrochilus japonius* was collected from the Sunqiao Park, Pudong New Area of Shanghai (121.64869°E; 31.18755°N), and the specimen was deposited at the Insect Laboratory of School of Wetlands, Yancheng Teachers University (Huicong Du: 174240355@qq.com) under the voucher number YUCT-20231010. The two samples were placed into anhydrous ethanol and returned to the laboratory, where they were stored at −20 °C for DNA extraction. A DNA extraction kit (Promega Corporation, Madison, WI, USA) was used to extract total DNA from the thoracic muscle tissue of *G. japonius*. A NanoDrop 2000 and Quantus Fluorometer (Thermo Fisher Scientific, Wilmington, DE, USA) were used to assess DNA purity (OD260/OD280 ratio) and concentration, respectively. After passing quality checks, the DNA samples were randomly fragmented using a Covaris M220 ultrasonic instrument (Covaris, Woburn, MA, USA), followed by whole library preparation through end repair, adapter ligation, purification. After the library was constructed, Qubit 3.0 and Qseq100 were used for preliminary quantification and quality assessment. Following confirmation of qualified library quality, different libraries were pooled into a flowcell, and cBOT-generated clusters were sequenced on an Illumina (HiSeq X), (Majorbio Bio-pharm Technology, Shanghai, China) yielding 4 Gb of raw sequence data. The coverage-depth map is provided in Figure S1. Trimmomatic software was used to trim the raw data and filter out low quality sequencing reads; the cleaned data were then assembled using the SPAdes (Dmitry et al. 2016). The mitoZ program (Meng et al. 2019) was used to assemble and annotate the mitochondrial genome.

### 2.2. Analysis of the characteristics and phylogenetic relationships of the mitogenome

MEGA 7.0.26 software (Paris, France) was used to calculate the nucleotide composition of the mitogenome, including the contents of C, G, A, and T bases, GC skew, AT skew, and codon usage for amino acid (Kumar et al. 2016). The secondary structures of tRNA genes were predicted by using tRNAscan-SE v2.0 (Lowe and Chan 2016). To clarify the phylogenetic placement of *G. japonius* within the family Nitidulidae, we constructed a maximum likelihood tree by analyzing 27 mitochondrial genomes of sap beetles from Nitidulidae, with *Monotoma quadricollis* Aube 1837 and *Rhizophagus aeneus* Richter used as outgroups from Monotomidae family, which have a close phylogenetic relationship with the Nitidulidae family (Table S1). The L-INS-i algorithm of the MAFFT v7.505 software was used for multiple sequence alignment, and then Gblocks v0.91b was employed to handle the gaps. The saturation analysis of the sequences was conducted using DAMBE v5.3.74. IQ-TREE software v2.2.0 was used for maximum likelihood analysis; the software automatically selected the optimal model as GTR+F + I + R5, and the node support values of the phylogenetic tree were evaluated using bootstrap analysis (1,000 replicates) (Minh et al. 2020).

## 3. Results

### 3.1. Mitochondrial genome characteristics of Glischrochilus japonius

**Figure 1.**
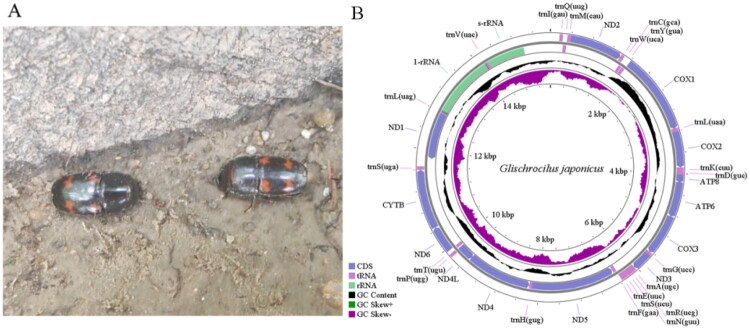
Species reference photograph (A: Photo by Jingxin Shen) and circular map of the complete mitogenome (B) of *Glischrochilus japonius*. The purple peaks indicated the deviation in GC-skew, and black peaks indicated the deviation of GC% divergence.

The complete length of the mitochondrial genome for *G. japonius* was 15,357 bp, consisting of 38 regions, including 13 protein-coding genes (PCGs), 22 tRNAs, 2 rRNAs, and 1 non-coding control region (Figure 1). There were 7 spacer regions between adjacent genes, among which the longest spacer is located between *trnS2* and *ND1* (158 bp). There were 19 overlapping genes, among which the overlap between *trnT* and *ND4L* is the longest (40 bp). And there were still 11 regions had no overlap or spacer (Table 1). Leu (count > 500) were the most used amino acid in PCGs, whereas His, Gln, Asp, Glu, Cys, Trp and Arg were the least used (count < 100) (Figure S2). UUU, UUA, AUU, and AUA (count > 200) had a relatively high frequency in codons, whereas CGC (count: 1) had the lowest frequency. Among the 22 tRNA genes in the mitogenome, 21 can form conventional cloverleaf structures, whereas tRNA^Ser(AGN)^ cannot form typical tRNA cloverleaf structures due to the absence of the D-arm (dihydrouridine arms). There were 21 base mismatches in the secondary structure of tRNA, including 16 G–U mismatches, 2 U–U mismatches, 1 A-A mismatch, and 2 C–A mismatches (Figure S3). The complete mitogenome contained 40.26%, 37.25%, 13.56%, and 8.93% of bases A, T, C, and G, respectively (Table 2). The content of A + T was 77.51%, and that of G + C was 22.49%.

**Table 1.** Organization of the mitochondrial genome of *Glischrochilus japonius.*

Gene	Direction	Start position	Stop position	Length	Anticodon	Start codon	Stop codon	Intergenic length
*trnI*	F	1	65	65	GAT			
*trnQ*	R	83	151	69	CAT			17
*trnM*	F	151	220	70	TTG			−1
*ND2*	F	221	1225	1005		ATC	TAA	0
*trnW*	F	1224	1291	68	TCA			−2
*trnC*	R	1291	1354	64	GCA			−1
*trnY*	R	1364	1429	66	GTA			9
*COI*	F	1430	2968	1539		ATG	TAA	0
*trnL2*	F	2964	3026	63	TAA			−5
*COII*	F	3027	3714	688		ATA	T	0
*trnK*	F	3715	3783	69	CTT			0
*trnD*	F	3784	3847	64	GTC			0
*ATP8*	F	3848	4003	156		ATT	TAG	0
*ATP6*	F	3997	4668	672		ATG	TAA	−7
*COIII*	F	4668	5452	785		ATG	TA	−1
*trnG*	F	5452	5515	64	TCC			−1
*ND3*	F	5513	5869	357		ATA	TAG	−3
*trnA*	F	5868	5933	66	TGC			−2
*trnR*	F	5933	5996	64	TCG			−1
*trnN*	F	5994	6057	64	GTT			−3
*trnS1*	F	6058	6124	67	GCT			0
*trnE*	F	6125	6193	69	TTC			0
*trnF*	R	6192	6256	65	GAA			−2
*ND5*	R	6260	7973	1714		ATA	T	3
*trnH*	R	7971	8034	64	GTG			−3
*ND4*	R	8034	9368	1335		ATG	TAA	−1
*ND4L*	R	9362	9691	330		TTG	TAG	−7
*trnT*	F	9652	9715	64	TGT			−40
*trnP*	R	9716	9778	63	TGG			0
*ND6*	F	9780	10286	507		ATA	TAA	1
*CytB*	F	10290	11429	1140		ATG	TAA	3
*trnS2*	F	11429	11496	68	TGA			−1
*ND1*	R	11655	12605	951		TTG	TAA	158
*trnL1*	R	12607	12671	65	TAG			1
*rrnL*	R	12634	13979	1346				−38
*trnV*	R	13959	14028	70	TAC			−21
*rrnS*	R	14029	14811	783				0
*CR*	F	14812	15357	546				0

**Table 2.** Base composition of the mitochondrial genomes of *Glischrochilus japonius.*

	Length (bp)	T %	A %	A + T %	AT-skew	C %	G %	C + G %	GC-skew
Whole genome	15357	37.25	40.26	77.51	0.04	13.56	8.93	22.49	−0.21
Protein coding genes (PCGs)	11179	43.08	33.21	76.29	−0.13	11.89	11.82	23.71	−0.03
tRNA	1451	37.49	40.32	77.81	0.04	9.44	12.75	22.19	0.14
rRNA	2129	39.41	42.79	82.20	0.04	11.88	5.92	17.80	−0.34

### 3.2. Phylogenetic relationship between Glischrochilus japonius and other sap beetles

**Figure 2.**
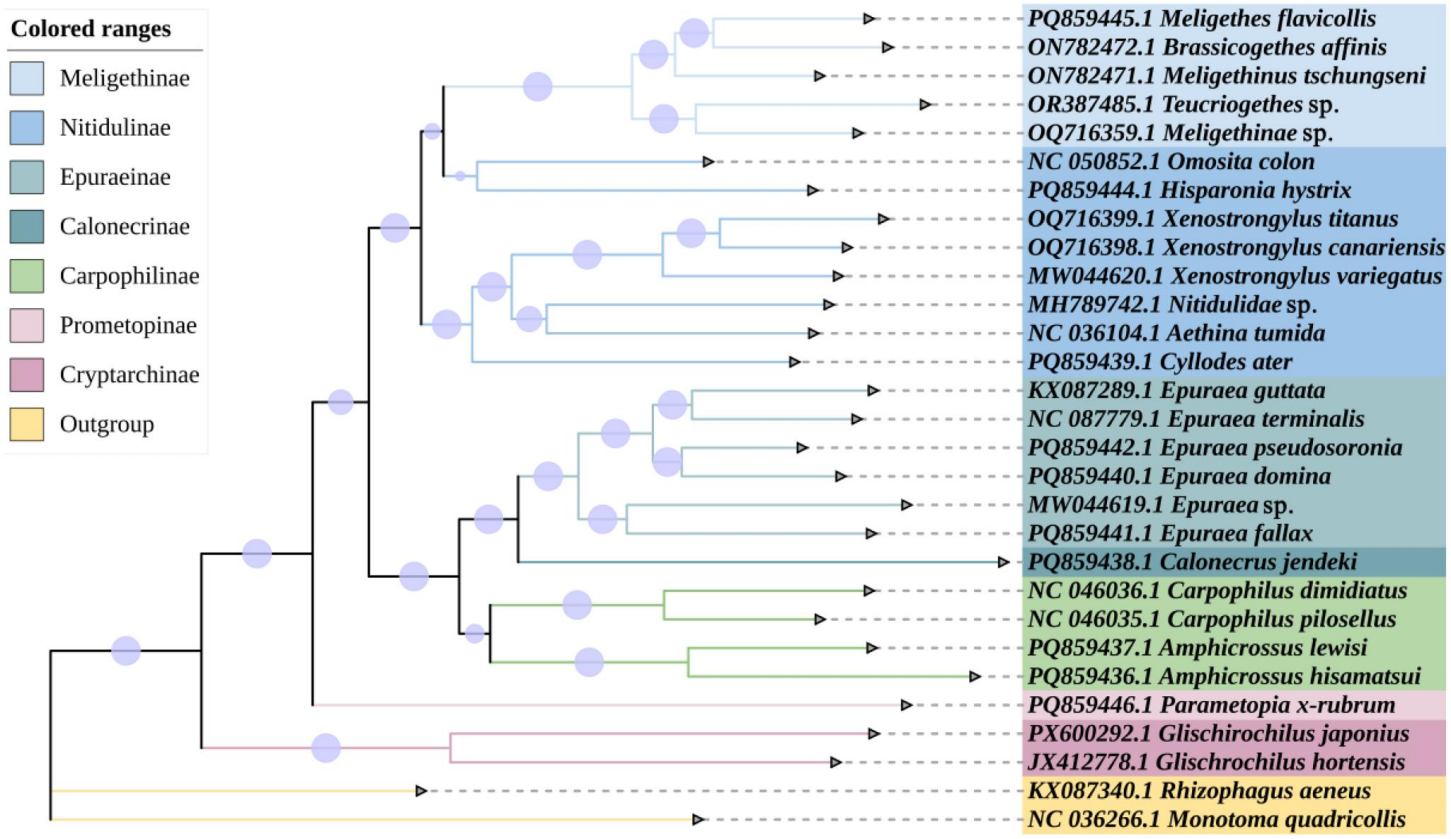
Phylogenetic tree constructed using representative beetle mitochondrial genomes using IQ-Tree. Boostrap support values are given at the nodes and indicated by circles sized for percentage. Different colored clades indicate different subfamilies and families.

Using *Monotoma quadricollis* and *Rhizophagus aeneus* as outgroups, the phylogenetic tree was constructed based on the mitochondrial genomes of 27 beetles to investigate the phylogenetic relationship of *G. japonius* with other species within Nitidulidae (Figure 2). The genera and subfamilies of the analyzed beetles are monophyletic in their phylogenetic relationships. The relationship between *G. japonius* and *Glischrochilus hortensis* (Fourcroy 1785) is relatively close. The phylogenetic relationships of each subfamily in Nitidulidae were: (((Meligethinae + Nitidulinae) + ((Epuraeinae +Calonecrinae) + Carpophilinae)) + Prometopinae) + Cryptarchinae. Most nodes were highly supported.

## 4. Discussion and conclusion

This study presents the complete mitochondrial genome of *G. japonius*, revealing the conservation of the mitogenome structure. The arrangement of protein-coding genes in the mitogenome of *G. japonius* is consistent with t*hat* reported for other sap beetles in Nitidulidae (Xu et al. 2021). The base composition of the mitochondrial genome was A > T > C > G, and the content of A + T is 77.51%, which is consistent with other sap beetles, such as *Omosita colon* (Xu et al. 2021) and *Meligethinus tschungseni* (Dai et al. 2024). With the exception of tRNA^Ser(AGN)^, which lacks the D-arm, the other 21 tRNAs exhibit classical cloverleaf structures, features also observed in other insect groups (Li et al. 2023; Su et al. 2023; Pang et al. 2024). The structural mismatch is predominantly caused by G–U pairs, which may occurs because mitochondrial genomes lack recombination and mutations or selection pressures during evolution (Chen et al. 2020; Su et al. 2023). The mismatch can be corrected by RNA editing, which does not hinder tRNA transport (Du et al. 2019). To analyze the phylogenetic relationships among sap beetles by constructing the maximum likelihood tree, we found that, in the current phylogenetic tree, genera and subfamilies of Nitidulidae are monophyletic. The results of the phylogenetic tree are supported by the results of Dai et al. (2024) and Chen et al. (2020). The current phylogenetic results show that G. japonius and *G. hortensis* are closely related, however, the definition and internal phylogeny of Nitidulidae remain in flux, and the phylogenetic relationships across the subfamily require further resolution. The mitochondrial genome data for Nitidulidae and other lower taxa (such as subfamilies and genera) are limited, and representative species from each genus or tribe cannot be widely selected for analysis, resulting in unbalanced datasets. Therefore, to obtain a more comprehensive and reliable phylogenetic reconstruction, a large number of mitogenomes representative of diverse species are needed.

## Supplementary Material

Supplementary Materials.pdf

## Data Availability

The genome sequence data that support the findings of this study are openly available in GenBank of NCBI at (https://www.ncbi.nlm.nih.gov/nuccore/PX600292.1) under the accession no. PX600292. The associated BioProject, Bio-Sample, and SRA numbers are PRJNA1308452, SAMN50695532, and SRR36288614 respectively.
