# Supplementary material for: The complete mitochondrial genome of Glischrochilus (Librodor) japonius (Coleoptera: Nitidulidae)
Source: Mitochondrial DNA B Resour. 2025 Dec 24;11(1):175–9. doi: 10.1080/23802359.2025.2606449 (PMC12777789; doi:10.1080/23802359.2025.2606449)

Table S1 The sequence information used for constructing the phylogenetic tree

Number	Species	GenBank number	literature resources
1	*Brassicogethes affinis*	ON782472.1	Dai et al., 2024
2	*Meligethinus tschungseni*	ON782471.1	Dai et al., 2024
3	*Nitidulidae* sp.	MH789742.1	Alex et al., 2015
4	*Aethina tumida*	NC_036104.1	Duquesne et al., 2017
5	*Xenostrongylus variegatus*	MW044620.1	Chen et al., 2020
6	*Meligethes flavicollis*	PQ859445.1	Unpublished
7	*Xenostrongylus titanus*	OQ716399.1	Unpublished
8	*Xenostrongylus canariensis*	OQ716398.1	Unpublished
9	*Cyllodes ater*	PQ859439.1	Unpublished
10	*Omosita colon*	NC_050852.1	Xu et al., 2021
11	*Hisparonia hystrix*	PQ859444.1	Unpublished
12	*Teucriogethes* sp.	OR387485.1	Unpublished
13	*Meligethinae* sp.	OQ716359.1	Unpublished
14	*Carpophilus dimidiatus*	NC_046036.1	Wu et al., 2020
15	*Carpophilus pilosellus*	NC_046035.1	Wu et al., 2020
16	*Amphicrossus lewisi*	PQ859437.1	Unpublished
17	*Amphicrossus hisamatsui*	PQ859436.1	Unpublished
18	*Calonecrus jendeki*	PQ859438.1	Unpublished
19	*Epuraea* sp.	MW044619.1	Chen et al., 2020
20	*Epuraea fallax*	PQ859441.1	Unpublished
21	*Epuraea guttata*	KX087289.1	Chen et al., 2020
22	*Epuraea terminalis*	NC_087779.1	Unpublished
23	*Epuraea pseudosoronia*	PQ859442.1	Unpublished
24	*Epuraea domina*	PQ859440.1	Unpublished
25	*Parametopia x-rubrum*	PQ859446.1	Unpublished
26	*Glischirochilus japonius*	PX600292.1	This study
27	*Glischrochilus hortensis*	JX412778.1	Unpublished
28	*Rhizophagus aeneus*	KX087340.1	Unpublished
29	*Monotoma quadricollis*	NC_036266.1	Unpublished

Figure S1. Read coverage plot across the *Glischrochilus japonius* mitogenome.

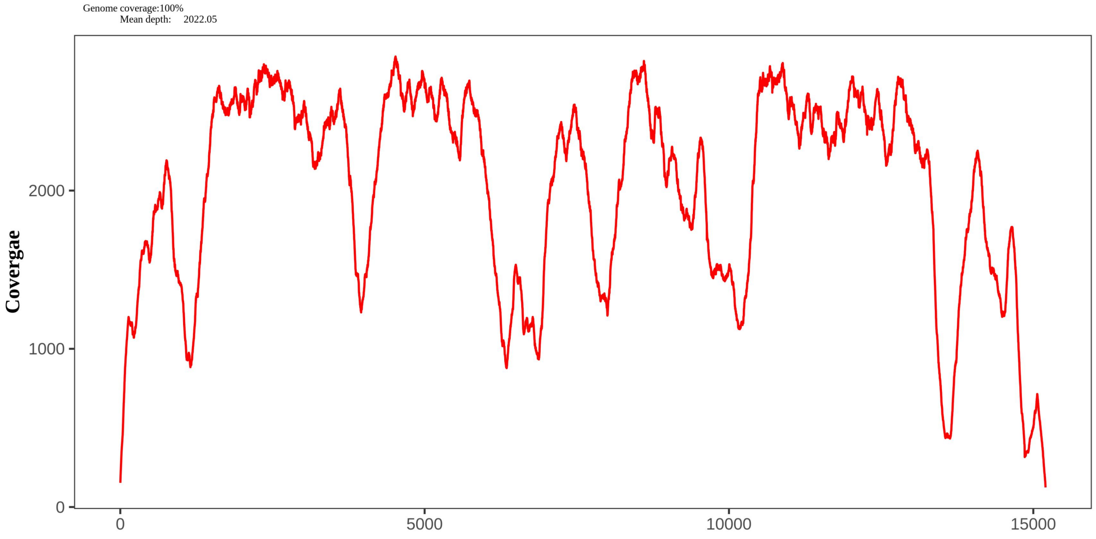

Figure S2. Codon usage of amino acid in *Glischrochilus japonius* mitogenome. Codon families in different color were provided on the horizontal axis, and codon usage were provided on the vertical

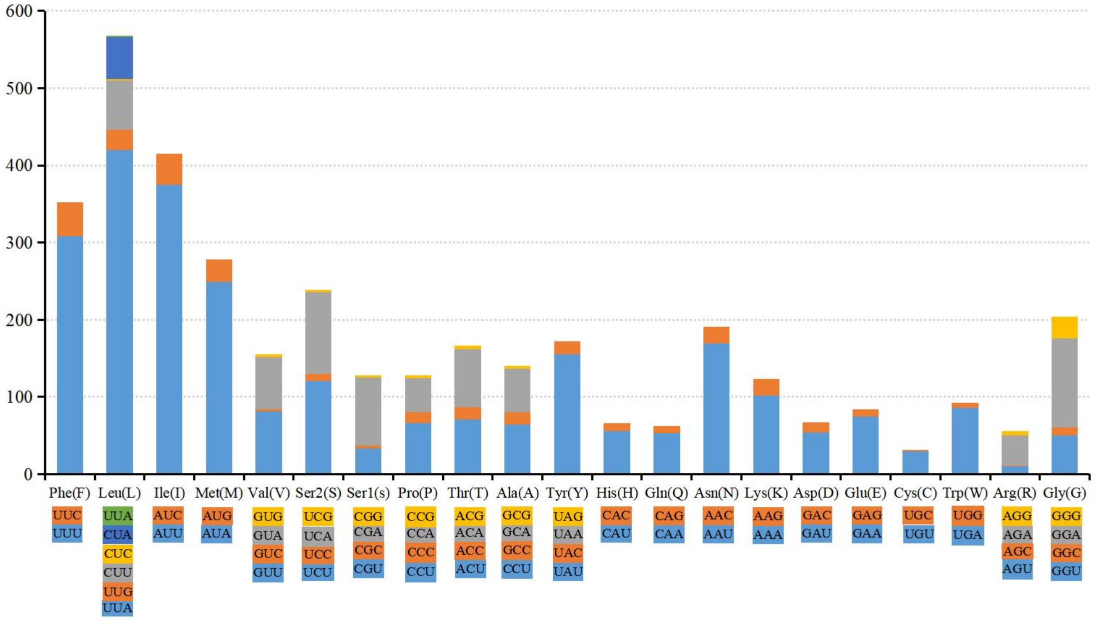

Figure S3 Secondary structures for tRNA genes from the mtDNA of *Glischrochilus japonius*

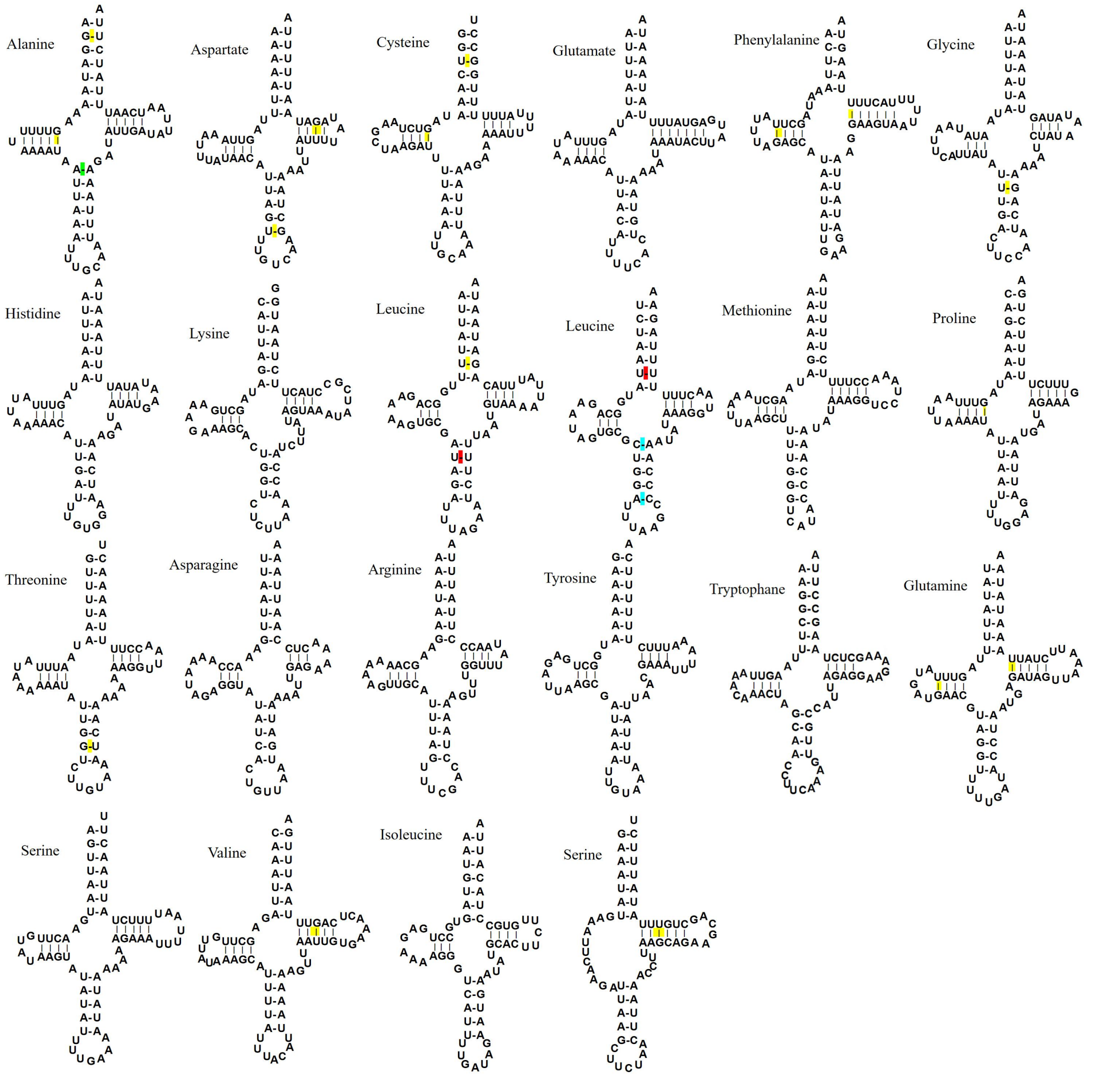

Supplement: Supplementary Materials.pdf [file TMDN_A_2606449_SM4473.pdf]
